# Standing under pressure: hemodynamic effects of abdominal compression type and intensity in healthy adults

**DOI:** 10.3389/fphys.2025.1621617

**Published:** 2025-08-20

**Authors:** Kishen Mitra, Sameer Kunte, Sara Taube, Shruthee Sankarlinkam, Liban Mohamed, Eghosa Adodo, Kevin A. Wu, Cynthia Green, Marat Fudim, Eric S. Richardson

**Affiliations:** 1 Department of Biomedical Engineering, Duke University, Durham, NC, United States; 2 Division of Cardiology, Duke University School of Medicine, Durham, NC, United States; 3 Department of Biostatistics and Bioinformatics, Duke University School of Medicine, Durham, NC, United States; 4 Duke Clinical Research Institute, Durham, NC, United States

**Keywords:** abdominal compression, orthostatic hemodynamics, surface area, active stand test, heart rate, blood pressure

## Abstract

**Introduction:**

Abdominal compression is recommended to manage orthostatic intolerance in dysautonomia, but the hemodynamic effects of different compression parameters remain poorly understood. This study investigated how surface area and pressure magnitude of abdominal compression affect blood pressure and heart rate responses during active stand tests in healthy volunteers. Understanding how abdominal compression modulates hemodynamics during standing in healthy individuals will help us better understand how compression can be optimized to benefit those with dysautonomia.

**Methods:**

Two compression devices were developed: one applying circumferential pressure (40 mmHg) over a higher surface area (HSA), and another applying focal pressure to the epigastrium at either 95 mmHg (LSA-LP) or 140 mmHg (LSA-HP). Forty-seven healthy participants completed randomized 3-min active stand tests with each device and a control condition. Heart rate was measured immediately upon standing (0 min) and at 15 s, 30 s, 1 min, 2 min, and 3 min afterward. Blood pressure was measured at 1-min intervals. All measurements were normalized to supine baseline values and presented as mean ± SEM.

**Results:**

All compression modalities significantly reduced the initial heart rate increase immediately upon standing compared to control (HSA: 2.0 ± 1.1 bpm, LSA-LP: 1.8 ± 1.0 bpm, LSA-HP: 2.7 ± 1.7 bpm vs. control: 6.0 ± 1.2 bpm; all p < 0.01). HSA compression showed greater hemodynamic effects than LSA-LP, with a significantly lower normalized heart rate at 0 min (p = 0.031). HSA compression was associated with higher systolic blood pressure compared to control at 3 min (7.2 ± 0.9 vs. 3.6 ± 0.9 mmHg; p = 0.006), and LSA-HP at 1 min (7.2 ± 1.0 vs. 3.8 ± 1.5 mmHg; p = 0.049). No significant differences were found between LSA-HP and LSA-LP across any timepoint.

**Discussion:**

Surface area appears to be a more critical factor than pressure magnitude in stabilizing hemodynamics during orthostatic stress, with significant effects observed immediately upon standing. These findings provide physiological insights for optimizing compression therapy in orthostatic disorders and suggest that wider-area compression garments may offer superior hemodynamic benefits compared to focal compression.

## 1 Introduction

Dysautonomia encompasses disorders of autonomic nervous system regulation, which controls involuntary functions including heart rate, blood pressure, and digestion (Wig and Oakley, 2019). One of the most common manifestations is orthostatic intolerance - the body’s inability to properly regulate cardiovascular function when transitioning from supine to upright position (Garland et al., 2015). Among the most challenging forms are postural orthostatic tachycardia syndrome (POTS) and neurogenic orthostatic hypotension (nOH), which significantly impact quality of life and daily function.

In healthy individuals, assumption of upright posture triggers compensatory mechanisms including increased heart rate, cardiac contractility, and vascular tone to maintain cerebral perfusion (Raj et al., 2005). Based on consensus criteria, POTS is characterized by an exaggerated heart rate response–specifically, a sustained increase of ≥30 beats/min (or ≥40 beats/min in individuals aged 12–19 years) within 10 min of upright posture, accompanied by orthostatic symptoms persisting for at least 3 months (Plash et al., 2013; Bourne et al., 2021). This occurs due to underlying hypovolemia, deconditioning, neuroendocrine dysfunction, and neuropathy (Gibbons et al., 2013), but without orthostatic hypotension (drop in blood pressure >20/10 mmHg) (Cai et al., 2021). In contrast, nOH is defined by inadequate sympathetic-mediated vasoconstriction leading to a sustained reduction in systolic blood pressure ≥20 mmHg or diastolic blood pressure ≥10 mmHg within 3 min of standing (Coniglio et al., 2022). Both conditions can manifest with symptoms including lightheadedness, palpitations, tremulousness, weakness, and impaired cognition that improve with recumbency (Stewart et al., 2007; Kaufmann et al., 2024).

Diagnosis of these conditions typically involves either head-up tilt tests (HUTs) or active stand tests (ASTs) to assess orthostatic responses. While HUTs have been traditionally used, ASTs provide a more physiologic assessment that better mimics daily activities (Finucane et al., 2019). Studies have shown that ASTs maintain comparable sensitivity to HUTs while offering improved specificity (Plash et al., 2013). This is partly due to the engagement of the skeletal muscle pump during active standing, which helps maintain venous return through compression of lower limb vessels (Cai et al., 2021).

While volume expansion and vasoconstrictive medications are mainstays of treatment, their effectiveness can be limited (Figueroa et al., 2010). For POTS, there remains a lack of FDA-approved medications (Miller and Raj, 2018), while nOH treatment options (midodrine and droxidopa) are often limited by side effects including supine hypertension (Okamoto et al., 2016). These therapeutic challenges highlight the need for effective non-pharmacological interventions.

Compression garments, such as abdominal binders and lower body compression garments extending at least to the xiphoid, can aid in preventing excessive venous pooling, thereby lessening symptoms in some patients (Figueroa et al., 2015; Okamoto et al., 2016; Fu and Levine, 2018; Junghans-Rutelonis et al., 2019). By promoting venous blood return to the heart, abdominal compression can help maintain adequate preload and cardiac output, which are essential for optimal cardiovascular function. Additionally, the activation of the celiac plexus and induction of vasoconstriction can contribute to the regulation of vascular tone and blood pressure, potentially improving hemodynamic stability (Arai et al., 2013; Lee et al., 2016). Both mechanisms have the potential to increase blood pressure and cardiac output and could thus be a valuable addition to existing POTS and nOH treatments. Transient improvements in cardiac output may prevent reflex tachycardia, which is characteristic of POTS.

However, traditional abdominal binders provide a fixed level of compression and may not offer adequate support to counteract the effects of orthostatic hypotension in cases where there is significant abdominal venous pooling (Mitra et al., 2024). Recent innovations have attempted to address these limitations. Okamoto et al. developed a servo-controlled inflatable binder showing comparable efficacy to midodrine (Okamoto et al., 2016). Previously, a group at our institution developed an air bladder inflation device that allows for manual adjustment of cuff pressure tailored to a patient’s needs (Coniglio et al., 2022). A 52-year-old man with orthostatic hypotension tolerated a pressure of around 50 mmHg and was able to ambulate starting from the first day of using the inflatable abdominal belt, leading to significant improvement in functional status (Coniglio et al., 2022).

While abdominal compression has been shown to be effective, there is little research on how this treatment modality can be optimized. Changing parameters such as surface area and magnitude of compression may create devices that better control underlying pathophysiology and symptoms in those with dysautonomia. Understanding the role of compression in dysautonomia pathology first requires clarifying its role in healthy physiology, an area with a paucity of literature. This study addresses this knowledge gap by investigating how two novel abdominal compression devices with different surface area configurations impact heart rate and blood pressure during standardized orthostatic testing. Additionally, this study evaluates how different magnitudes of pressure impact heart rate and blood pressure during standardized orthostatic testing.

## 2 Materials & methods

### 2.1 Subjects

Forty-seven participants were recruited between October 2023 and April 2024. Participants were included if they were between the ages of 18 and 64 years. Participants were screened for eligibility via REDCap electronic data capture tools hosted at Duke University that were accessible from QR code on recruitment flyers (Harris et al., 2009).

Exclusion criteria included inability to give informed consent, hypertension (>150/100 mmHg during screening or in accordance with medical history), current pregnancy, existing history of cardiovascular diseases or dysautonomia, and past medical history of medical implants (i.e., pacemakers). The protocol was reviewed and approved by the Duke University Institutional Review Board (IRB Protocol 2024-0056).

### 2.2 Compression devices

The study evaluated two distinct abdominal compression devices. The first, called the “circumferential pressure device” (abbreviated as “HSA” for high surface area), employs an inflatable air bladder integrated into a broad, stiff waist belt to apply circumferential pressures of up to 40 mmHg. This device utilized the same form factor as previously reported in our group’s previous nOH patient case report (Coniglio et al., 2022).

The second device, called the “focal pressure device” (abbreviated as “LSA” for low surface area), employs a harness with an integrated piston capped with a flat 6.45 cm^2^ plastic disc. Using an air compressor, the piston extrudes to apply pressure to the user’s epigastrium. The device is secured to the participant’s upper abdomen via plastic frame and cord ties. Study personnel apply counterforce to the piston head while switching to the “on” position to prevent forceful abdominal contact, then slowly release counterforce for gradual engagement.

Both devices were internally tested to ensure consistent, safe, and reproducible pressure delivery throughout the experimental period. Calibrated pressure gauges were used to validate that each device would provide their target pressure over the span of 3 min without significant variability. These procedures were repeated multiple times for each device to confirm reliability and consistency in pressure application.

### 2.3 General protocol

Testing was performed at our laboratory between 8 a.m. and 5 p.m. in a quiet environment with stable ambient temperature of 22°C–24°C. All participants provided written informed consent prior to participation and had abstained from consuming alcohol or tobacco for 24 h and caffeine for 6 h before participation. Participants were requested to avoid wearing compressive clothing such as high-waisted leggings or pants to prevent additional abdominal compression from clothing.

Information on age, sex, race, height, and weight were collected on all participants. Throughout testing, participants wore a commercially available brachial blood pressure cuff (GE DURA-CUF) and SpO_2_ probe (Masimo) on the contralateral arm. Data from these devices were recorded on a GE Dash 4,000 patient monitor that displayed heart rate (HR) continuously, and systolic blood pressure (SBP) and diastolic blood pressure (DBP) intermittently as described below. Pulse pressure (PP) was derived by subtracting SBP from DBP at each measurement timepoint. Before beginning trials, participant abdominal girth was measured at the umbilicus using a tape measure.

### 2.4 Active stand test (AST)

A randomized crossover treatment study design was used, with each participant completing a series of ASTs, each of 3-min duration, with 3 min of rest between each AST. The 3-min duration was selected based on established protocols for assessing short-term neural and cardiovascular function as a convenient mimic of daily activities (Finucane et al., 2019). Initially, the study protocol included three conditions: no compression (Control), higher surface area compression (HSA), and lower surface area with low-magnitude pressure compression (LSA-LP). As the study progressed, a fourth condition was added to the protocol: lower surface area with high-magnitude pressure compression (LSA-HP).

The first AST was always conducted with no compression device as a control (abbreviated as “Control”). Participants were then randomized into subsequent ASTs using the two study devices. One AST was completed with the circumferential pressure device (HSA) and two ASTs with the focal pressure device, one with low pressure (95 mmHg, abbreviated as “LSA-LP”) and one with high pressure (140 mmHg, abbreviated as “LSA-HP”). To reduce donning and doffing times, trials with the point pressure device were completed in series, randomizing whether high or low pressure would be applied first.

**FIGURE 1:**
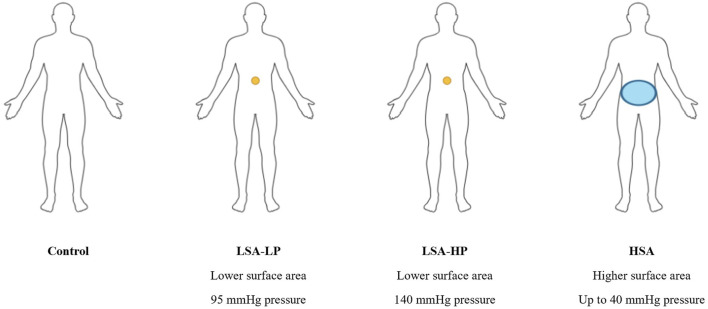
Compression device configirations used in AST. Representation of the compression conditions: small surface area with lower magnitude of pressure (LSA-LP), small surface area with higher magnitude of pressure (LSA-HP), and circumferential pressure across a higher surface area (HSA). Each participant completed four 3-min active stand tests (ASTs) with each of the four compression conditions, in a semi-randomized order.

Each AST began with the participant lying supine to allow their HR and BP to equilibrate. For ASTs using an abdominal compression device, the device would be disengaged during the supine period to avoid applying pressure to the abdomen. After 3 min, the abdominal compression device, if worn, would be engaged to apply pressure and the participant would then rise to a standing position for 3 minutes (Figure 1). Standing followed a pre-determined protocol that was practiced with the participants prior to the trials.

HR was measured upon standing and at 15 s, 30 s, 1 min, 2 min, and 3 min after standing; SBP and DBP were measured upon standing and at 1-min intervals after standing. During all trials, participants were asked to not speak and to be as still as possible as not to distort readings.

### 2.5 Statistical methods

Baseline characteristics and baseline (supine) hemodynamic parameters are presented as mean (standard deviation [SD]) and median (25th-75th percentiles [Q1-Q3]) for continuous variables and as frequencies and percentages for categorical variables. The distribution of data was assessed using Shapiro-Wilk normality tests. Baseline (supine) hemodynamic parameters across the four treatments (Control, LSA-LP, LSA-HP, and HSA) were compared using Kruskal–Wallis tests, as these raw data were not normally distributed.

For each participant, the change (delta) in SBP, DBP, PP, and HR from supine baseline to standing was calculated at each measurement timepoint and presented as the mean ± standard error of the mean (SEM).

Primary analyses focused on comparing the effects of each compression treatment (HSA, LSA-HP, and LSA-LP) to the Control treatment on delta SBP, DBP, PP, and HR. Repeated measures analysis of variance (ANOVA) models adjusted for baseline value, time, treatment, and time-by-treatment interaction were used to examine differences at each specific timepoint during standing, as well as to evaluate differences across the entire standing period. The empirical covariance (“sandwich”) estimator was used to provide a robust variance estimate with compound-symmetry structure. Secondary analyses examined: (1) the effect of compression surface area by comparing the higher surface area treatment (HSA) to both lower surface area treatments (LSA-HP and LSA-LP), and (2) the effect of pressure magnitude by comparing the higher pressure treatment (LSA-HP) versus lower pressure treatment (LSA-LP).

For all comparisons, p-values for repeated measures models are presented without adjustment for multiple testing. Post-hoc adjustment for multiple pairwise comparisons using the Benjamini–Hochberg false discovery rate (FDR) are presented where necessary. A significance level of α = 0.05 was used for all statistical tests. Data were exported from REDCap and imported into SAS version 9.4 (SAS Institute, Inc., Cary, NC) for statistical analyses.

### 2.6 Sample size and power analysis

While previous studies have examined compression effects in patient populations, no data were available regarding differential cardiovascular responses to focal versus global abdominal compression in healthy adults. Based on pilot testing and previous compression literature, we estimated that a clinically meaningful difference between compression modalities would be a 5 mmHg change in systolic blood pressure or a 5 bpm difference in heart rate, with an estimated standard deviation of 8 mmHg and 7 bpm, respectively. Using these conservative estimates, for a repeated measures design with four conditions and a 0.05 two-sided significance level, a sample size of 43 participants would provide at least 90% power to detect these differences. We recruited 47 participants to account for potential incomplete data collection or dropouts.

## 3 Results

### 3.1 Demographics and baseline characteristics

A total of 47 individuals participated in this study with a mean age of 24.0 (SD 2.8) years and 63.8% (n = 30) female (Table 1). The racial distribution comprised 55.3% (n = 26) Asian/Pacific Islander, 34.0% (n = 16) White, 8.5% (n = 4) Black, and 2.1% (n = 1) Hispanic. Average abdominal girth measured at the navel was 79.3 cm (SD 9.4), average weight was 65.2 kg (SD 13.7), and average BMI was 22.6 kg/m^2^ (SD 3.1). All 47 participants completed the AST under control conditions (no device) and with HSA compression. Of these, 46 completed testing with LSA-LP compression and 21 with LSA-HP compression.

**TABLE 1:** Baseline characteristics of the study cohort.

	Total (N = 47)
Age (years)	
Mean (SD)	24.0 (2.8)
Range	19–29
Gender, n (%)	
Male	17 (36.2%)
Female	30 (63.8%)
Race, n (%)	
White/Caucasian	16 (34.0%)
Black or African American	4 (8.5%)
Hispanic	1 (2.1%)
Asian/Pacific Islander	26 (55.3%)
Weight (kg)	
Mean (SD)	65.2 (13.7)
Median (Q1-Q3)	65.8 (57.2–72.6)
Abdominal Girth (cm)	
Mean (SD)	79.3 (9.4)
Median (Q1-Q3)	76 (72–87)
BMI (kg/m^2^)	
Mean (SD)	22.6 (3.1)
Median (Q1-Q3)	22.3 (20.4–24.4)

During each AST, participant BP and HR were collected while supine with abdominal compression devices disengaged when present. These supine measurements served as a baseline against which BP and HR data collected during the same AST were normalized. No statistically significant differences between treatment conditions at baseline were identified for SBP, DBP, or PP. However, baseline supine HR differed significantly across treatments (p = 0.025), with median (Q1-Q3) values ranging from 59 (55-64) bpm in the LSA-HP condition up to 69 (59-76) bpm in the control (Table 2).

**TABLE 2:** Measurements at baseline (supine) across different compression conditions.

	Control (N = 47)	HSA (N = 47)	LSA-HP (N = 21)	LSA-LP (N = 46)	P-value
SBP					0.68
Median (Q1-Q3)	110 (102–117)	106 (101–119)	106 (99–120)	110 (104–118)	
Mean (SD)	111.5 (11.6)	109.7 (11.5)	110.6 (12.5)	110.2 (11.8)	
DBP					0.54
Median (Q1-Q3)	59 (57–63)	59 (57–63)	57 (55–60)	58 (57–64)	
Mean (SD)	61.1 (7.3)	60.7 (6.5)	60.8 (6.9)	60.7 (7.0)	
PP					0.72
Median (Q1-Q3)	51 (45–55)	47 (43–53)	47 (43–57)	47 (43–55)	
Mean (SD)	50.4 (7.8)	48.9 (10.2)	48.5 (8.7)	49.7 (9.2)	
HR					0.025
Median (Q1-Q3)	69 (59–76)	66 (56–74)	59 (55–64)	62 (57–71)	
Mean (SD)	68.3 (11.2)	65.9 (12.1)	60.5 (7.8)	63.4 (9.6)	

Groups compared using the Kruskal–Wallis test.

Abbreviations: SBP, systolic blood pressure; DBP, diastolic blood pressure; PP, pulse pressure; HR, heart rate; SD, standard deviation.

### 3.2 Device effects

The three study treatments were compared to control to determine their impact on BP and HR during AST, with analyses focusing on differences at specific timepoints as well as across the entire standing period.

#### 3.2.1 Control vs. HSA compression

HSA compression showed no significant effect (mean ± SEM) on normalized SBP compared to control until the 3-min timepoint, where HSA was associated with a significantly higher SBP (7.2 ± 0.9 mmHg vs. 3.6 ± 0.9 mmHg; p = 0.006) (Table 3). No significant differences in normalized DBP or PP were observed at any timepoint. Notably, HSA compression significantly attenuated the initial HR increase upon standing, with a mean change of −2.0 ± 1.1 bpm compared to 6.0 ± 1.2 bpm in the control condition (p < 0.001). This HR-stabilizing effect was not sustained at subsequent timepoints. When analyzing the entire standing period using repeated measures ANOVA, no significant differences were found between control and HSA for any hemodynamic parameter, though SBP approached significance (p = 0.08) (Supplementary Table S7). Overall, HSA was associated with a significantly lower initial normalized HR and a significantly higher 3-min normalized SBP than control.

**TABLE 3:** Hemodynamic changes for Control vs. Higher Surface Area Compression.

Timepoint	Control	HSA	Control vs. HSA p-value
ΔHR, bpm			
0 min	6.0 ± 1.2	−2.0 ± 1.1	**<0.001** ^a^
0.25 min	24.2 ± 2.0	22.4 ± 2.2	0.27
0.5 min	7.3 ± 1.7	6.8 ± 1.7	0.55
1 min	11.6 ± 1.6	11.6 ± 1.5	0.79
2 min	15.1 ± 1.5	13.9 ± 1.4	0.32
3 min	15.2 ± 1.5	16.7 ± 1.4	0.64
ΔSBP, mmHg			
0 min	10.0 ± 1.3	11.4 ± 1.4	0.49
1 min	5.0 ± 0.9	7.2 ± 1.0	0.13
2 min	4.6 ± 1.0	6.3 ± 1.0	0.25
3 min	3.6 ± 0.9	7.2 ± 0.9	**0.006**
ΔDBP, mmHg			
0 min	8.8 ± 0.9	9.4 ± 0.9	0.60
1 min	6.9 ± 0.6	8.3 ± 1.0	0.23
2 min	7.7 ± 0.7	8.9 ± 0.9	0.33
3 min	7.6 ± 0.8	8.8 ± 0.9	0.31
ΔPP, mmHg			
0 min	1.2 ± 1.1	2.0 ± 1.1	0.70
1 min	−1.9 ± 0.8	−1.1 ± 1.0	0.66
2 min	−3.2 ± 0.9	−2.6 ± 1.1	0.79
3 min	−4.0 ± 1.0	−1.6 ± 1.1	0.14

Values are mean ± standard error of the mean (SEM). Repeated measures ANOVA, compared differences between the no compression (Control) and circumferential compression (HSA) tests. Bold values indicate a statistically significant difference between Control and HSA, without adjustment for multiple testing. Bold indicates significant value (p < 0.05).

^a^ Remained significant (p < 0.001) after post-hoc adjustment using Benjamini–Hochberg false discovery rate (FDR).

ΔSBP, change in systolic blood pressure; ΔDBP, change in diastolic blood pressure; ΔPP, change in pulse pressure; ΔHR, change in heart rate; ANOVA, analysis of variance.

#### 3.2.2 Control v LSA-HP compression

Similar to HSA, LSA-HP significantly reduced the initial HR increase upon standing (2.7 ± 1.7 bpm vs. 6.0 ± 1.2 bpm; p = 0.009), with no significant differences at later timepoints (Table 4). No significant differences in normalized SBP, DBP or PP were observed between conditions. Repeated measures ANOVA across the entire standing period revealed no significant differences between control and LSA-HP for any hemodynamic parameter (Supplementary Table S7). Overall, LSA-HP was associated with a significantly lower initial normalized HR than control.

**TABLE 4:** Hemodynamic changes for Control vs. Lower Surface Area with High Magnitude Pressure Compression.

Timepoint	Control	LSA-HP	Control vs. LSA-HP p-value
ΔHR, bpm			
0 min	6.0 ± 1.2	2.7 ± 1.7	**0.009**
0.25 min	24.2 ± 2.0	23.6 ± 3.1	0.38
0.5 min	7.3 ± 1.7	11.2 ± 2.0	0.58
1 min	11.6 ± 1.6	15.6 ± 1.9	0.44
2 min	15.1 ± 1.5	20.7 ± 1.4	0.10
3 min	15.2 ± 1.5	21.3 ± 1.6	0.068
ΔSBP, mmHg			
0 min	10.0 ± 1.3	9.4 ± 1.7	0.69
1 min	5.0 ± 0.9	3.8 ± 1.5	0.42
2 min	4.6 ± 1.0	4.8 ± 1.5	0.96
3 min	3.6 ± 0.9	5.3 ± 1.2	0.31
ΔDBP, mmHg			
0 min	8.8 ± 0.9	9.0 ± 1.4	0.90
1 min	6.9 ± 0.6	6.8 ± 1.3	0.87
2 min	7.7 ± 0.7	7.4 ± 1.6	0.84
3 min	7.6 ± 0.8	7.0 ± 1.5	0.71
ΔPP, mmHg			
0 min	1.2 ± 1.1	0.4 ± 1.3	0.50
1 min	−1.9 ± 0.8	−3.0 ± 1.7	0.36
2 min	−3.2 ± 0.9	−2.7 ± 1.7	0.90
3 min	−4.0 ± 1.0	−1.7 ± 1.5	0.23

Values are mean ± standard error of the mean (SEM). Repeated measures ANOVA, compared differences between the no compression (Control) and focal compression with high magnitude of pressure (LSA-HP) tests. Bold values indicate a statistically significant difference between Control and LSA-HP, without adjustment for multiple testing.

ΔSBP, change in systolic blood pressure; ΔDBP, change in diastolic blood pressure; ΔPP, change in pulse pressure; ΔHR, change in heart rate; ANOVA, analysis of variance. Bold indicates significant value (p < 0.05).

#### 3.2.3 Control v LSA-LP compression

LSA-LP compression demonstrated no significant impact on normalized SBP, DBP, or PP at any timepoint compared to control (Table 5). However, consistent with other compression modalities, LSA-LP significantly attenuated the initial HR increase upon standing (1.8 ± 1.0 bpm vs. 6.0 ± 1.2 bpm; p < 0.001), with no significant differences observed at subsequent timepoints. Analysis of the entire standing period showed no significant differences between control and LSA-LP for any parameter (Supplementary Table S7). Overall, LSA-LP was associated with a significantly lower initial normalized HR than control.

**TABLE 5:** Hemodynamic changes for Control vs. Lower Surface Area with Low Magnitude Pressure Compression.

Timepoint	Control	LSA-LP	Control vs. LSA-LP p-value
ΔHR, bpm			
0 min	6.0 ± 1.2	1.8 ± 1.0	**<0.001** ^a^
0.25 min	24.2 ± 2.0	25.8 ± 1.7	0.91
0.5 min	7.3 ± 1.7	9.0 ± 1.5	0.93
1 min	11.6 ± 1.6	13.1 ± 1.3	0.95
2 min	15.1 ± 1.5	17.1 ± 1.3	0.78
3 min	15.2 ± 1.5	18.9 ± 1.4	0.22
ΔSBP, mmHg			
0 min	10.0 ± 1.3	7.9 ± 1.2	0.22
1 min	5.0 ± 0.9	5.7 ± 1.0	0.65
2 min	4.6 ± 1.0	6.0 ± 1.1	0.36
3 min	3.6 ± 0.9	5.3 ± 1.8	0.24
ΔDBP, mmHg			
0 min	8.8 ± 0.9	8.6 ± 0.8	0.88
1 min	6.9 ± 0.6	7.1 ± 0.7	0.86
2 min	7.7 ± 0.7	8.2 ± 0.7	0.65
3 min	7.6 ± 0.8	8.3 ± 0.8	0.59
ΔPP, mmHg			
0 min	1.2 ± 1.1	−0.7 ± 1.1	0.18
1 min	−1.9 ± 0.8	−1.4 ± 0.9	0.73
2 min	−3.2 ± 0.9	−2.2 ± 1.1	0.54
3 min	−4.0 ± 1.0	−2.9 ± 1.0	0.44

Values are mean ± standard error of the mean (SEM). Repeated measures ANOVA, compared differences between the no compression (Control) and focal compression with lower magnitude of pressure (LSA-LP) tests. Bold values indicate a statistically significant difference between Control and LSA-LP, without adjustment for multiple testing. Bold indicates significant value (p < 0.05).

^a^ Remained significant (p = 0.012) after post-hoc adjustment using Benjamini–Hochberg false discovery rate (FDR).

ΔSBP, change in systolic blood pressure; ΔDBP, change in diastolic blood pressure; ΔPP, change in pulse pressure; ΔHR, change in heart rate; ANOVA, analysis of variance.

### 3.3 Effects of surface area

To determine the effect of compression surface area on hemodynamic parameters, we compared the HSA compression device to both configurations of the LSA device.

#### 3.3.1 HSA v LSA-HP

HSA compression produced a significantly higher normalized SBP than LSA-HP at 1 min after standing (7.2 ± 1.0 mmHg vs. 3.8 ± 1.5 mmHg; p = 0.049) (Supplementary Table S6). Conversely, LSA-HP was associated with a significantly higher normalized HR at 2 min compared to HSA (20.7 ± 1.4 bpm vs. 13.9 ± 1.4 bpm; p = 0.004). No other significant differences in normalized SBP, DBP, or PP were observed at any individual timepoint. When analyzing the entire standing period, no significant differences were detected between HSA and LSA-HP for any hemodynamic parameter (Supplementary Table S7). Overall, HSA was associated with a significantly higher 1-min normalized SBP and a significantly lower 2-min normalized HR than LSA-HP.

#### 3.3.2 HSA v LSA-LP

No significant differences in normalized SBP, DBP, or PP were detected between HSA and LSA-LP devices across any timepoint (Supplementary Table S6). However, LSA-LP was associated with a significantly higher normalized HR immediately after standing compared to HSA (1.8 ± 1.0 bpm vs. −2.0 ± 1.1 bpm; p = 0.031), with no significant differences at later timepoints. Repeated measures ANOVA for the entire standing period revealed no significant differences between HSA and LSA-LP for any parameter (Supplementary Table S7). Overall, HSA was associated with a significantly lower initial normalized HR than LSA-LP.

### 3.4 Effects of pressure magnitude

To assess whether pressure magnitude influenced hemodynamic responses, we compared LSA-HP and LSA-LP compression conditions.

#### 3.4.1 LSA-HP v LSA-LP

No significant differences in normalized SBP, DBP, PP, or HR were observed between LSA-HP and LSA-LP conditions at any individual timepoint (Supplementary Table S6). Similarly, repeated measures ANOVA across the entire standing period showed no significant differences between LSA-HP and LSA-LP for any parameter (Supplementary Table S7). Overall, there were no significant differences between LSA-HP and LSA-LP.

### 3.5 Temporal hemodynamic response patterns

**FIGURE 2:**
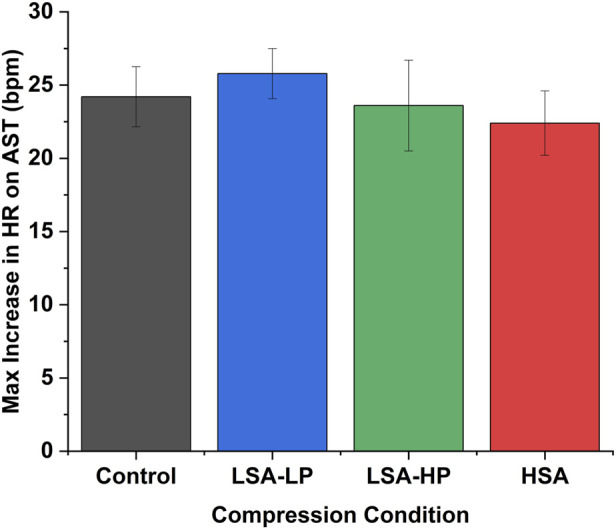
Maximum heart rate increase during active stand test across compression conditions. Data represent peak HR changes occurring at t = 0.25 min for all conditions (mean ± SEM). HSA demonstrated the lowest maximum HR increase (22.4 ± 2.2 bpm) compared to Control (24.2 ± 2.0 bpm), though differences between compression conditions did not reach statistical significance.

Figure 2 illustrates the temporal patterns of normalized hemodynamic responses across treatment conditions over the 3-min standing period. All compression modalities consistently demonstrated attenuation of the initial HR increase upon standing, with varying effects on blood pressure parameters (Figure 2).

**FIGURE 3:**
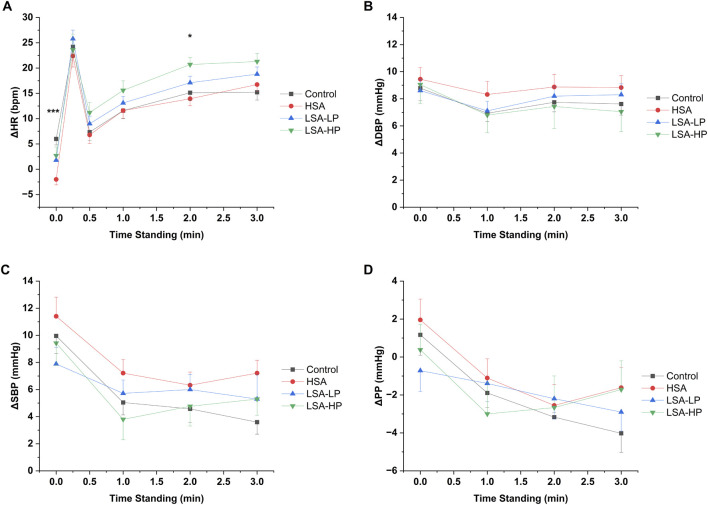
Hemodynamic responses for each of the compression conditions. **(A)** Change in heart rate (ΔHR). **(B)** Change in systolic blood pressure (ΔSBP). **(C)** Change in diastolic blood pressure (ΔDBP). **(D)** Change in pulse pressure (ΔPP) from supine to standing 3 min. Data are mean ± SEM. *p < 0.05, **p < 0.01, ***p < 0.001 indicate significant difference between treatment groups from repeated measures ANOVA.

The maximum HR increase occurred consistently at t = 0.25 min across all conditions (Figure 3). HSA compression demonstrated the lowest maximum HR increase (22.4 ± 2.2 bpm) compared to Control (24.2 ± 2.0 bpm), LSA-LP (25.8 ± 1.7 bpm), and LSA-HP (23.6 ± 3.1 bpm), though these differences did not reach statistical significance (p = 0.57).

The temporal evolution of blood pressure parameters varied by treatment, with HSA compression maintaining higher SBP at later timepoints compared to control (Figure 2), while LSA compression configurations showed more modest effects on blood pressure components throughout the observation period. Notably, HSA and LSA-HP displayed similar pulse pressure temporal profiles (Figure 2), both trending less negative than control at the 3-min timepoint. Despite these observed trends, repeated measures ANOVA across the entire standing period revealed no statistically significant effects of any compression modality on overall hemodynamic patterns compared to control (Supplementary Table S7).

## 4 Discussion

This study investigating the effects of surface area and magnitude of abdominal compression on BP and HR in healthy individuals during ASTs has three main findings. First, abdominal compression blunted physiologic HR increases shortly upon standing with some delayed effects on BP. Second, a larger surface area of abdominal compression led to greater effects on BP and HR. Finally, the magnitude of pressure applied during low surface area compression had minimal effects on BP and HR.

### 4.1 Cardiovascular adaptations during postural change

The physiology underlying hemodynamic changes in ASTs have been well described. Transitioning from supine to standing requires the contraction of skeletal muscle groups in the lower extremities and abdomen (Borst et al., 1982). This triggers the exercise reflex causing a transient reduction in vagal tone that is responsible for an increase in HR seen 0–5 s after standing (Freyschuss et al., 2025). Once standing, significant shifts in blood volume occur due to gravity, causing nearly 700 mL of venous pooling in the abdomen and lower extremities (Shibao et al., 2013). Impaired venous return, or preload, causes a decline in cardiac output and arterial pressure. As a result, SBP and DBP can decline up to 20 mmHg within 10 s of standing (van Wijnen et al., 2017). Declines in arterial pressure reduce the baroreflex causing a second increase in HR seen around 5–12 s after standing (Imholz et al., 1990). Increased sympathetic tone due to reduced arterial pressure also causes an increase in cardiac contractility and peripheral vasoconstriction leading to overshoot and stabilization of BP near to supine BP within 30 s of standing (Finucane et al., 2019). BP overshoot causes HR to decline and stabilize at a rate greater than supine HR.

### 4.2 Hemodynamic stabilization through compression

All four ASTs led to a similar trend in normalized HR after standing: they peaked around 20–25 bpm at 15 s (Figure 3), fell to around 10 bpm at 30 s, and stabilized from 1 to 3 min. The key difference was observed immediately after standing, where compression treatments reduced normalized HR by 4-8 bpm compared to control. This suggests that abdominal compression helps maintain venous return and cardiac output by preventing venous pooling, thereby reducing the need for compensatory HR increases. Although all compression modalities demonstrated some reduction in the peak HR response at 15 s, these effects did not reach statistical significance, suggesting that certain aspects of the baroreflex response remained intact despite improved hemodynamics.

The effects of abdominal compression on BP were mixed. Both HSA and LSA-HP had delayed effects on BP with HSA increasing SBP at 3 min and LSA-HP decreasing SBP at 1 min. It is unclear why disparate effects were seen in different types of abdominal compression. It is possible that the increased surface area of HSA compression continues to improve preload and cardiac output throughout standing, resulting in increased SBP at 3 min. Conversely, LSA-HP applied high pressure to the epigastrium which may have caused transient inhibition of the celiac plexus. The celiac plexus provides sympathetic innervation to the vasculature of the abdomen. Pharmacologic block of this structure has been shown to decrease blood pressure (Arai et al., 2013; Lee et al., 2016). A transient inhibition of the celiac plexus via LSA-HP may have impaired vasoconstriction in the abdomen, leading to blunted arterial overshoot seen around 1 min after standing.

A trial by Tanaka et al. using compression stockings of different lengths in healthy participants during head-up tilt (HUT) found similar results. Participants wearing waist-high stockings that provided abdominal compression had a reduction in the transient HR increases after HUT (Tanaka et al., 2014). Thigh-high and knee-high stockings did not have the same effect, indicating the importance of abdominal compression. Of note, this study found no significant difference in normalized arterial pressure for the 120 s measured after HUT.

### 4.3 Surface area drives hemodynamic response

The primary difference between the two compression devices used is the surface area of applied pressure. Given the average abdominal girth of participants (79.3 cm), the HSA device applies compression over approximately 800 cm^2^. Meanwhile, the LSA device applies compression over only 6.25 cm^2^.

Compared to LSA-LP, HSA compression blunted HR increases by 3.8 bpm upon standing up. This is significant as LSA-LP was shown to prevent HR increases compared to control, indicating a probable relationship between surface area of compression and effect on immediate HR changes due to standing. The larger surface area of HSA compression likely led to significantly less venous pooling than LSA-LP, leading to improvements in cardiac output and an attenuated HR increase. The change in venous pooling and preload was likely not large enough to result in BP differences between the devices.

There was not a significant difference in HR immediately after standing between HSA and LSA-HP, indicating that both treatments similarly reduce venous pooling. HSA, however, improved SBP at 1 min and reduced HR at 2 min compared to LSA-HP. The difference in SBP may be explained by the previously proposed mechanism of LSA-HP inhibiting the celiac plexus leading to reduced abdominal vasoconstriction. These reductions in abdominal vascular tone may have caused the reflexive HR increase seen at 2 min in LSA-HP.

### 4.4 Pressure magnitude shows limited effects

The LSA-HP and LSA-LP trials differed in their magnitude of pressure applied (140 vs. 95 mmHg, respectively). No differences in normalized SBP, DBP, PP, or HR were seen when directly comparing the two ASTs. Interestingly, there was a difference in HR immediately after standing when comparing HSA and LSA-LP but not when comparing HSA and LSA-HP. As both LSA-HP and LSA-LP have identical surface areas, this may indicate that the increased magnitude of pressure used in LSA-HP led to a greater reduction in venous pooling. More data may be needed to clarify the relationship between magnitude of compression and hemodynamics after standing.

While the hemodynamics of ASTs in healthy participants have been well studied, there is a lack of literature on the influence of abdominal compression. Furthermore, this study is the first to analyze the effects of surface area and magnitude of compression. Understanding how these parameters impact healthy individuals is crucial to improving methods of abdominal compression for the treatment of pathology like POTS.

### 4.5 Study limitations and considerations

A limitation of this study was the lack of older participants. Participant ages ranged from 19 to 29 years old. While the hemodynamic response to AST is largely similar amongst healthy individuals, there are some crucial changes that occur with age. Around the sixth decade of life, HR increases seen immediately after standing begin to blunt (Dambrink and Wieling, 1987). This age-related change implies that older participants may respond less to abdominal compression than their younger counterparts. Another limitation of this study was the lack of continuous blood pressure and electrocardiographic monitoring. BP was measured at 1-min intervals after standing. While we found that abdominal compression affected SBP and DBP, using a beat-to-beat monitor may have revealed transient changes in BP that we were unable to capture. Additionally, continuous electrocardiographic monitoring would have allowed us to evaluate heart rate variability, an important indicator of autonomic nervous system activity. The final limitation was the lack of hemodynamic measures like cardiac stroke volume, venous return velocity, and vascular resistance. With these measures, we would be able to directly confirm how abdominal compression impacts hemodynamics rather than making assumptions based on HR and BP changes.

### 4.6 Implications for future compression therapy development

This investigation represents a proof-of-concept study that systematically examines how different compression parameters affect orthostatic hemodynamics in healthy individuals. Our findings establish a physiological foundation for understanding the mechanisms by which compression garments might benefit patients with orthostatic intolerance. The observation that surface area appears more influential than pressure magnitude has significant implications for compression therapy design.

These results serve as important benchmarks that enhance our understanding of compression therapy mechanisms and can help optimize device effectiveness in clinical practice. For patients with orthostatic disorders such as POTS or nOH, our findings suggest that garments providing widespread abdominal compression may be more effective than focal compression devices, even when the latter apply higher pressures.

The next logical step is to assess whether these same relationships hold true in patients with dysautonomia, where autonomic dysfunction alters normal cardiovascular compensatory mechanisms. Additionally, these findings may have broader clinical applications, such as optimizing compression strategies for hemodynamic stabilization during surgery and critical care settings, where maintaining venous return and cardiac output is essential.

## 5 Conclusion

This study provides new insights into how compression surface area and pressure magnitude influence hemodynamic responses during orthostatic stress. Our findings demonstrate that surface area appears to be a more critical factor than pressure magnitude in stabilizing cardiovascular responses to standing. This knowledge offers a physiological basis for optimizing compression garment design for orthostatic intolerance and highlights the importance of considering compression parameters beyond simple pressure measurements. As we move forward in addressing the needs of patients with orthostatic disorders, these findings provide an evidence-based foundation for developing more effective compression strategies tailored to specific clinical requirements.

## Data Availability

The raw data supporting the conclusions of this article will be made available by the authors, without undue reservation.
