# Correction: Standing under pressure: hemodynamic effects of abdominal compression type and intensity in healthy adults

**DOI:** 10.3389/fphys.2025.1718352

**Published:** 2025-11-24

**Authors:** Kishen Mitra, Sameer Kunte, Sara Taube, Shruthee Sankarlinkam, Liban Mohamed, Eghosa Adodo, Kevin A. Wu, Cynthia Green, Marat Fudim, Eric S. Richardson

**Affiliations:** 1 Department of Biomedical Engineering, Duke University, Durham, NC, United States; 2 Division of Cardiology, Duke University School of Medicine, Durham, NC, United States; 3 Department of Biostatistics and Bioinformatics, Duke University School of Medicine, Durham, NC, United States; 4 Duke Clinical Research Institute, Durham, NC, United States

**Keywords:** abdominal compression, orthostatic hemodynamics, surface area, active stand test, heart rate, blood pressure

Author Kevin A. Wu was omitted as an author in the published article. The correct **author list** reads:

Kishen Mitra^1,†^, Sameer Kunte^2,†^, Sara Taube^1,†^, Shruthee Sankarlinkam^1^, Liban Mohamed^2^, Eghosa Adodo^2^, Kevin A. Wu^2^, Cynthia Green^3,4^, Marat Fudim^2,4^, Eric S. Richardson^1,*^

^1^Department of Biomedical Engineering, Duke University, Durham, NC, United States

^2^Division of Cardiology, Duke University School of Medicine, Durham, NC, United States

^3^Department of Biostatistics and Bioinformatics, Duke University School of Medicine, Durham, NC, United States

^4^Duke Clinical Research Institute, Durham, NC. United States

† These authors contributed equally to this work and share first authorship

The **Author Contributions** Statement has been corrected to read:

KM: Conceptualization, Investigation, Methodology, Software, Writing – original draft, Writing – review and editing. SK: Conceptualization, Data curation, Investigation, Methodology, Software, Writing – original draft, Writing – review and editing. ST: Conceptualization, Investigation, Methodology, Writing – original draft, Writing – review and editing. SS: Investigation, Methodology, Writing – review and editing. LM: Investigation, Writing – review and editing. EA: Investigation, Writing – review and editing. KW: Investigation, Writing – review and editing. CG: Formal Analysis, Writing – review and editing. MF: Investigation, Validation, Writing – review and editing. ER: Conceptualization, Methodology, Validation, Writing – review and editing.

The original article has been updated.

