# Supplementary material for: Standing under pressure: hemodynamic effects of abdominal compression type and intensity in healthy adults
Source: Front Physiol. 2025 Aug 20;16:1621617. doi: 10.3389/fphys.2025.1621617 (PMC12405206; doi:10.3389/fphys.2025.1621617)
Supplement: Supplementary file 1 [file Supplementaryfile1.docx]

Supplementary Material

# Supplementary Figures and Tables

## Supplementary Tables

**Supplemental Table 6.** Pairwise comparisons for each of the compression conditions. Data are presented as the mean ± SEM and compared using a repeated measures ANOVA initially without adjustment for multiple testing. C = Control, LSA-LP = Lower surface area with lower magnitude of pressure, LSA-HP = Lower surface area with higher magnitude of pressure, HSA = Circumferential pressure across higher surface area. *Pairwise comparisons that remained statistically significant (p<0.05) after post-hoc adjustment using Benjamini-Hochberg false discovery rate (FDR).

| **Parameter** | **Phase** | **Control (C)** | **LSA-LP** | **LSA-HP** | **HSA** |  | **Overall** |  | **C v LSA-LP** | **C v**  **LSA-HP** | **C v HSA** | **HSA vs LSA-HP** | **HSA vs LSA-LP** | **LSA-HP vs LSA-LP** |
| --- | --- | --- | --- | --- | --- | --- | --- | --- | --- | --- | --- | --- | --- | --- |
| **Heart Rate**  **(bpm)** | **0 min** | 6.0 ± 1.2 | 1.8 ± 1.0 | 2.7 ± 1.7 | -2.0 ± 1.1 |  | **<0.001** |  | **<0.001*** | **0.009** | **<0.001*** | 0.10 | **0.031** | 0.97 |
|  | **0.25 min** | 24.2 ± 2.0 | 25.8 ± 1.7 | 23.6 ± 3.1 | 22.4 ± 2.2 |  | 0.57 |  | 0.91 | 0.38 | 0.27 | 0.98 | 0.28 | 0.41 |
|  | **0.5 min** | 7.3 ± 1.7 | 9.0 ± 1.5 | 11.2 ± 2.0 | 6.8 ± 1.7 |  | 0.72 |  | 0.93 | 0.58 | 0.55 | 0.26 | 0.46 | 0.60 |
|  | **1 min** | 11.6 ± 1.6 | 13.1 ± 1.3 | 15.6 ± 1.9 | 11.6 ± 1.5 |  | 0.75 |  | 0.95 | 0.44 | 0.79 | 0.28 | 0.71 | 0.42 |
|  | **2 min** | 15.1 ± 1.5 | 17.1 ± 1.3 | 20.7 ± 1.4 | 13.9 ± 1.4 |  | **0.038** |  | 0.78 | 0.10 | 0.32 | **0.004*** | 0.15 | 0.12 |
|  | **3 min** | 15.2 ± 1.5 | 18.8 ± 1.4 | 21.3 ± 1.6 | 16.7 ± 1.4 |  | 0.24 |  | 0.22 | 0.068 | 0.64 | 0.12 | 0.39 | 0.45 |
| **Systolic Blood Pressure (mmHg)** | **0 min** | 10.0 ± 1.3 | 7.9 ± 1.2 | 9.4 ± 1.7 | 11.4 ± 1.4 |  | 0.28 |  | 0.22 | 0.69 | 0.49 | 0.32 | 0.058 | 0.50 |
|  | **1 min** | 5.0 ± 0.9 | 5.7 ± 1.0 | 3.8 ± 1.5 | 7.2 ± 1.0 |  | 0.22 |  | 0.65 | 0.42 | 0.13 | **0.049** | 0.30 | 0.25 |
|  | **2 min** | 4.6 ± 1.0 | 6.0 ± 1.1 | 4.8 ± 1.5 | 6.3 ± 1.0 |  | 0.58 |  | 0.36 | 0.96 | 0.25 | 0.33 | 0.86 | 0.42 |
|  | **3 min** | 3.6 ± 0.9 | 5.3 ± 1.8 | 5.3 ± 1.2 | 7.2 ± 0.9 |  | 0.058 |  | 0.24 | 0.31 | **0.006** | 0.18 | 0.22 | 0.89 |
| **Diastolic Blood Pressure (mmHg)** | **0 min** | 8.8 ± 0.9 | 8.6 ± 0.8 | 9.0 ± 1.4 | 9.4 ± 0.9 |  | 0.91 |  | 0.88 | 0.90 | 0.60 | 0.79 | 0.48 | 0.80 |
|  | **1 min** | 6.9 ± 0.6 | 7.1 ± 0.7 | 6.8 ± 1.3 | 8.3 ± 1.0 |  | 0.67 |  | 0.86 | 0.87 | 0.23 | 0.46 | 0.31 | 0.97 |
|  | **2 min** | 7.7 ± 0.7 | 8.2 ± 0.7 | 7.4 ± 1.6 | 8.9 ± 0.9 |  | 0.76 |  | 0.65 | 0.84 | 0.33 | 0.42 | 0.56 | 0.64 |
|  | **3 min** | 7.6 ± 0.8 | 8.3 ± 0.8 | 7.0 ± 1.5 | 8.8 ± 0.9 |  | 0.64 |  | 0.59 | 0.71 | 0.31 | 0.27 | 0.60 | 0.45 |
| **Pulse Pressure**  **(mmHg)** | **0 min** | 1.2 ± 1.1 | -0.7 ± 1.1 | 0.4 ± 1.3 | 2.0 ± 1.1 |  | 0.31 |  | 0.18 | 0.50 | 0.70 | 0.30 | 0.081 | 0.56 |
|  | **1 min** | -1.9 ± 0.8 | -1.4 ± 0.9 | -3.0 ± 1.6 | -1.1 ± 1.0 |  | 0.67 |  | 0.73 | 0.36 | 0.66 | 0.24 | 0.90 | 0.26 |
|  | **2 min** | -3.2 ± 0.9 | -2.2 ± 1.1 | -2.7 ± 1.7 | -2.6 ± 1.1 |  | 0.94 |  | 0.54 | 0.90 | 0.79 | 0.93 | 0.75 | 0.72 |
|  | **3 min** | -4.0 ± 1.0 | -2.9 ± 1.0 | -1.7 ± 1.5 | -1.6 ± 1.1 |  | 0.44 |  | 0.44 | 0.23 | 0.14 | 0.92 | 0.42 | 0.56 |

**Supplemental Table 7.** P-values shown are obtained from repeated measures ANOVA to evaluate differences across devices for the entire standing period, as well as pairwise differences between devices across the entire standing period with no adjustment for multiple testing. C = Control, LSA-LP = Lower surface area with lower magnitude of pressure, LSA-HP = Lower surface area with higher magnitude of pressure, HSA = Circumferential pressure across higher surface area.

| **Parameter** | **Overall** | **C v LSA-LP** | **C v**  **LSA-HP** | **C v HSA** | **HSA vs LSA-HP** | **HSA vs LSA-LP** | **LSA-HP vs LSA-LP** |
| --- | --- | --- | --- | --- | --- | --- | --- |
| **Heart Rate**  **(bpm)** | 0.84 | 0.75 | 0.90 | 0.10 | 0.10 | 0.14 | 0.66 |
| **Systolic Blood Pressure (mmHg)** | 0.23 | 0.77 | 0.90 | 0.08 | 0.13 | 0.19 | 0.72 |
| **Diastolic Blood Pressure (mmHg)** | 0.69 | 0.90 | 0.73 | 0.52 | 0.42 | 0.63 | 0.68 |
| **Pulse Pressure**  **(mmHg)** | 0.28 | 0.94 | 0.93 | 0.43 | 0.46 | 0.49 | 0.88 |
